# Sulphate of Zinc in Hemorrhages

**Published:** 1858-11

**Authors:** Wm. Hauser

**Affiliations:** Speir’s Turnout, Ga.


### ARTICLE II.

*Sulphate of Zinc in Hemorrhages.* By WM. HAUSER, M. D., of Speir's Turnout, Ga.

My mind has been directed to this subject by reading, in the September number of the *Journal*, Dr. Thurman's article on "Hemorrhage from the extraction of teeth."

At the outset of my medical career, fifteen years ago, I one day asked my preceptor, Dr. Sam'l B. Clark of Brothersville, Ga., (Heaven's blessings attend him wherever he goes! No nobler man or better physician than he honors and blesses any country,) what he would do in a case of excessive bleeding from extracting a tooth. "I would use a solution of Sulphas Zinci," said he; "and I'll give you a case in point; I extracted a tooth for old bro. ———, the blood gushed forth alarmingly; I flew to my saddle-bags, dashed the Zinc and some water together, poured his mouth full of it, it was soon in a *pucker*, as if he had been eating green persimmons, and the hemorrhage was instantly and effectually stopped."

On that item of instruction, I have based many a successful treatment of bleeding, and that too without any special regard to the chemical condition of the blood; though as Dr. Thurman hints, this point merits close attention by those who would pursue an enlightened and not an empirical course of treatment. When the coagulum of the blood is in a normal condition very little difficulty is ordinarily experienced in arresting hemorrhages, as is well known, save when large blood vessels are involved; but very different is the case when all the coagulability of the blood has been destroyed and it comes rushing from one of its broken conduits, like water from a ruptured engine hose. But even in such a condition of blood as is here indicated, I have found nothing equal in styptic power to the Sulphate of Zinc dissolved in cold water. Nothing can be more readily convenient for use than it, for every physician has, or ought to have, a full supply of it in his saddle-bags wherever he may be; and nothing is to be done except to throw a teaspoonful of this salt into a teacup half full of cold water, and it is ready for use. I have found it just the thing for Epistaxis, in cases too numerous to mention. One I'll give: In 1843 a boy aged 13, addicted to nose bleeding, suffered for half a night in a room adjoining mine, his poor mother all the while working with all her power and skill to stop the hemorrhage; cold water, cotton wadding, magic, charms, all the mighty machinery of the blood-stoppers which she had learned from old grannies, negroes, witches, horse doctors and what not, failed utterly, and the boy appeared fast hastening to the spirit land; his stingy unprincipled father lying all the time in bed, and neither attempting to help his wife a particle, nor asking me to do it. I could stand it no longer, the boy must not bleed to death because his father was too mean to pay for treating him; I sprang from bed, and, as quickly as possible, injected a small syringe full of the solution in question, up his nose, instantly every bleeding capillary shut its mouth, and all was peace and hope again; a second return, and a second course of treatment, if memory be correct, and his hemorrhage ceased, to return no more while he lived in Georgia.

Case 2d: E. O. B. aged 19; now a promising young lawyer in Dublin, Ga., was fast going off with Dysentery; all styles of treatment pursued variously for two or three days had failed to stop the bleeding of his rectum. I charged him heavily with my favorite styptic, it caused severe pain, but the hemorrhage was stopped, to return no more. His convalescence was rapid, although his system had become enfeebled even before his attack.

In uterine hemorrhages, when other modes of treatment fail, I use this, tho' I hesitate long on account of the pain it is sure to produce when applied to a bleeding mucous surface.

I much admire the boldness, originality and skill displayed by Dr. Moyer, of Talbotton, Ga., in treating that inveterate case of uterine hemorrhage with Nitras Argenti.

